# Social gradient in health-related quality of life among urban middle-age residents in Limassol, Cyprus: research article

**DOI:** 10.1186/s12889-020-10027-6

**Published:** 2021-03-29

**Authors:** P. Ellina, N. Middleton, E. Lambrinou, C. Kouta

**Affiliations:** Department of Nursing, Faculty of Health Sciences, Cyprus University of Technology, 30 Archbishop Kyprianou Str, 3036 Limassol, Cyprus

**Keywords:** Social inequalities, Quality of health, SF 36, Social status, Gender

## Abstract

**Background:**

Social inequalities in health threaten social cohesion and their investigation is an important research field. Monitoring the health of the population is necessary to identify health needs, design programs focused in people’s needs and to evaluate the effectiveness of health policies.

**Methods:**

A cross-sectional survey using primary data was applied. The study investigated the size and the extent of social inequalities in quality of life and health behaviours in Limassol, Cyprus. Data collection was done door-to-door in the form of survey interviews. The sample consisted of 450 residents aged 45–64 across 45 randomly selected neighbourhoods, that met the selection criteria. The tools used were: Demographic questionnaire, SF 36 Questionnaire, IPAQ- International Physical Activity Questionnaire short form.

**Results:**

The social gradient appears in all social indicators. Physical dimension of health has a strong relationship between health-related quality of life with the education index. Specifically, the range is 12 points for males and 14 points for females (p for interaction = 0.16). Profession systematically appears to have a stronger relationship with men than with women, and is present in both physical and mental dimensions. The range is 13 points for men and 10 points for women (p for interaction = 0.31).

**Conclusions:**

It seems that young highly educated males, employed full time, earning high income and engaging in mild physical activity, have significantly higher level of health-related life quality, compared to other middle age adult groups, living in Limassol. This finding is in agreement with other studies that show correlations between gender and the patterns of risk factors.

**Supplementary Information:**

The online version contains supplementary material available at 10.1186/s12889-020-10027-6.

## Background

Social inequalities in health refer to systematic differences in health between different socio-economic groups. When these characteristics are compared, the difference is noticeable between what one would perceive as a simple fluctuation in health and social inequality in health. When the observed differences are systematic, they are considered as the effect of unbalanced social processes (which are modifiable) and an indication of unfairness [1].

Social inequalities in health threaten social cohesion. Poverty reduction, provision of effective health care to citizens and improvement of the quality of life, are long-term goals ensuring social and economic cohesion [2–4].

Social inequalities in health have been widely investigated. Researchers on health inequalities in recent decades have devoted considerable effort to identifying specific social, environmental or behavioral factors (e.g. occupational status, physical activity) that explain the relationship between social status and health [5–7]. The environment where one lives, plays a crucial role in this matter. However, the existence of socio-economic disadvantage, psychosocial effects (social cohesion), health behavior and gender can partially explain the social classification of health [4, 6–9].

It is generally accepted that no single factor can explain these inequalities in health, due to the combined and accumulative effect of risk factors and in different life domains [10]. It also seems that male and female are exposed differently or that they are otherwise vulnerable to these determinants [11–13]. Women have lower mortality rates but, paradoxically, report higher levels of depression, psychiatric disorders and various chronic diseases than men [10, 12, 13].

Usually, there is a gradual, if not even linear, decreasing trend in the health status. The lower the social status of the individual - this is not simply the case where poor health is confined to a single social group at the extreme end of the scale, while all other groups have relatively good health levels - is referred to as social gradient [1].

Sometimes, the impact of the social gradient in health is expressed as shortfall - which basically is expressed as the number of lives that would not have been lost if all groups in society enjoyed the same standard of life, as those in the most advantageous position [1]. For example, the shortfall in life expectancy for women in the lowest income group was around 26.7 years, compared to women - belonging to the highest income group [14].

In addition, there is increasing interest in monitoring the quality of life through perceived health status. This has been shown through longitudinal studies related to the provision for subsequent hospitalization or mortality [15–18].

Monitoring population’s health is vital for several reasons. This is necessary to identify health needs, design programs and to evaluate the effectiveness of health policies [15].

### Impact in community nursing and public health

The existence of social inequalities in health is a challenge for public health. At the same time, public health is a core of work for all nurses. Nurses view the effects of social inequalities on the health and well-being of their communities, such as inadequate access to health care services, increased morbidity and mortality rates. Because nurses experience the impact of social determinants on health, both- the patients they provide care for and the general population, have a clear stake in identifying and addressing the causes of “poor” health. Empowering people to get control of their lives, will help considerably to take control of their health and, having nurses as allies [19].

The results of this study can be utilized by community nurses, such as applying approaches aimed at reducing health inequalities, ensuring health and well-being, ensuring the effectiveness of initiatives and enhancing local authorities, in order to fulfil their obligations.

Community actors and local authorities should take into account the results of the research in order to take action in areas with the lowest values in people’s quality of life by implementing programs aimed at ensuring the well-being of the general population.

## Methods

### Aim

The purpose of this paper was to investigate the size and the extent of social inequalities in quality of life and health behaviours in Limassol.

### Design

This is a cross-sectional survey using primary data.

### Study participants

#### Sampling method

Multistage sampling was performed in four stages - parishes and communities, neighbourhoods (specific streets as a starting point from all parishes), homes (households), and finally, people.

- For municipalities and communities, a stratified random sampling was used in terms of urban areas, community size and generally accepted socio-economic indicators, to ensure that municipalities/communities from the entire range of socioeconomic classification were selected. More specifically, parishes and communities were ranked in terms of the percentage of the population with university education, an indicator which is one of the most commonly accepted socio-economic indicators and was the only one available at the stage of sampling from the open access files of the Cyprus Statistical Service.
- Neighborhoods: In order to select neighborhoods from the whole range of socio-economic scale, as well as, to achieve wider geographical coverage, it was decided to include streets from all the parishes of the city of Limassol.
- Households: The households that participated in the study were selected in a systematic way. In the city, the field researcher started from one end of the road and moved along its entire length, choosing houses along the entire length of the street, on the left and right side of the road alternately. Where it was not possible to recruit 10 people by the end of the selected street (e.g. refusal to participate, no one is home, etc.), the researcher completed the sample with households from the wider neighborhood - which is defined in the case of study as all lanes beginning/ending or crossing the selected street until the next intersection. In the case of an apartment building, a similar practice of systematic sampling based on the floors and the apartments was applied (e.g. left apartment on the 1st floor, right apartment on the 3rd floor accordingly avoiding repeating the same pattern).
- People: Quota sampling was used at household level (50:50, male female alternately in each household). The medical history of each person who participated in the research was not asked, as long as the person could answer the questions in the questionnaire. When the researcher had the required numbered, she stopped, but she was trying to get people from the beginning, middle and end of the road.

#### Sample size

The population of the city of Limassol was divided into quarters, depending on its socio-economic position and specifically on the percentage of the population with university education.

The estimation of sample was based on power analysis. The result size in this population group in the city of Limassol is f = 0.20–0.25 (the which corresponds to a moderate effect size). The sample size of 188–300 individuals provides 90% statistical power to detect a difference in statistical significance level of 5%. The minimum desired sample in urban areas was set at 450 people. The sample size ensures a similar level of statistical power to detect such a degree social gradient in the quality of life in both genders separately, at least in urban areas (225 men and 225 women). Finally, it should be noted that, due to the nature of the study, which is based on multi-stage random sampling of neighbourhoods/communities and households, it was taken into account that the sample size should also ensure the greatest possible geographical coverage and include neighbourhoods with diverse socioeconomic background. In terms of the objectives of the study, it is preferable in such cases that the sample consists of as many neighbourhoods/communities as possible rather than selecting many people from a small number of neighbourhoods and communities, so that the sample is more representative of the whole range of socio-economic disadvantage, which is expected to be concentrated in the area. The minimum number of people per neighbourhood/area was set at 10 people.

Further, it should be mentioned that the specific sample size is considered satisfactory as it provides statistical accuracy of ±5 percentage points for the 95% confidence interval in the estimation of percentages (e.g. smoking).

In order to ensure a satisfactory response rate, postal communication (distribution of an open letter to all homes on the preselected roads) informed prospective participants that a university researcher would visit them for a short interview in the next few days. Information was also given on the importance and contribution of the study with the request to participate.

### Inclusion criteria

- People aged 45 to 65, as to include middle age adults as the economically active population who has completed their studies, has a family, income, and therefore has been integrated into their own professional, income and social status. The choice of the 45–65 age group was based on two popular hypotheses, which seek to explain the mechanisms that lead to the effect of socio-economic position on health in old age. These hypotheses are: The “cumulative disadvantages / advantages” hypothesis and the “age as a leveler” hypothesis [20]. Health inequalities appear to be smaller at younger ages, wider at middle and early older ages, and smaller again at later ages [21].
- People who can speak and read in Greek
- Permanent residents in Limassol (or permanent resident or residence in Limassol for 5 years)

### Tools

#### Demographic / socio-economic characteristics and lifestyle characteristics

The demographic questionnaire used was developed for this study and includes variables related to personal characteristics, such as demographic characteristics (age, gender, marital status, area where they reside), socio-economic characteristics (level of education, annual income, occupation), as well as lifestyle characteristics (smoking, alcohol, physical activity) [Additional file 1: Appendix 1].

#### Self-rated health

The level of self-assessment of the individual’s health was measured on a 5-point scale (Likert scale), ranging from excellent health to poor. Individuals were asked to evaluate their health as: 1 = excellent, 2 = very good, 3 = good, 4 = moderate and 5 = poor.

#### SF 36 questionnaire - quality of life

Quality of life questionnaire SF-36v2 Standard, Greek Version was used to measure quality of life of research participants. The questionnaire was created in 1992 [22], and is used in several countries for the self-esteem of the Quality of Life and comparing the health status of different population groups.

The SF-36 scale is a tool used to measure the health level of a population. Its basic attribute is the simultaneous measurement and assessment of the level of physical and mental health. The grid of 36 questions includes eight measurement scales consisting of questions that represent the most measured health dimensions. These scales are: physical functioning (PF), role physical (RP), bodily pain (BP), general health (GH), vitality (VT), social functioning (SF), role emotional (RE), and mental health (MH). The first four (4) dimensions make up the physical health, while other mental health of the individual. These eight scales are evaluated with a score ranging from 0 to 100 each, where 0 represents the minimum possible value and at the same time the worst health, while the maximum score of the scale, the value 100 is excellent health. Where a score of less than 50 this means that the person’s health is below the average [23]. The generality of the SF-36 questions allows the adaptability of the questionnaire to each group of the population, while the Greek translation, as well as the entire questionnaire have been tested in repeated surveys in the health sector in Greece [24, 25] and Cyprus [26, 27].

#### IPAQ- international physical activity questionnaire short form

The International Physical Activity Questionnaire short form (IPAQ) is a popular and frequently used questionnaire, which was developed in the late ‘90s by a multinational working group, supported by the World Health Organization, to be used for comparative evaluation of physical population activity different groups and nationalities [28]. The IPAQ-short is also used by the European Union (Eurobarometer), while it has been tested in many international studies and is characterized by high reliability and satisfactory validity [28].

#### Social status and indicators of socio-economic disadvantage

The social status of the individual will be measured by the level of education, occupation, income.

Educational measurement refers to the highest level of education an individual attended (None / Primary, Secondary-Lower, Secondary-Upper, Undergraduate, Postgraduate studies).

Regarding the occupation, because the Cyprus Statistical Service classifies occupations only in the field of employment- which does not concern graduation, the Standard Occupational Classification of United Kingdom was used. Each participant was categorized according to his / her occupation.

The income indicator refers to the classification of individuals in relation to the monthly family income and was assessed according to the Cyprus Statistical Service standard through categories in which each participant was asked to choose one.

### Data collection procedure

For the data collection door-to-door survey method was employed. The survey was oral, in the form of an interview. The interviewer asked all participants the same set of questions and completed all sections of the questionnaire on paper during the interview. The interviewer visited each address once. Four hundred fifty residents aged 45–64 (50:50 gender quota) across 45 randomly selected neighbourhoods (10 systematic random sample per area) from different city quarters, stratified by population density and proportion of adult residents with tertiary education.

The data collected wherever the participant wanted, whether it was inside the house or at the door. The questionnaire filling time was about 10 min.

The data collection was mainly done in the afternoon, as during these hours target group was more likely to be at home. In case of refusal to participate, the frequency (number of people who said they did not want to participate) was counted, to have an overview of the response rate (losses).

### Ethical considerations

An opinion was sought from the Cyprus National Bioethics Committee and a reply has been received that this investigation does not fall within the remit of the CNBC for further bioethical evaluation. The participants, who wished to participate in the research, gave oral consent. In addition, anonymity and confidentiality of information were ensured, as only the researcher, research assistants and supervising professors had access. The questionnaires were destroyed after analyzing the data.

### Statistical analysis

Statistical analysis was performed using the statistical package IBM SPSS Statistics 23 and the significance level was set at *p* < 0,05.

The mean value (M) and standard deviation (SD) were used to describe quantitative variables. Data concerning the International Physical Activity Questionnaire short form (I-PAQ) was entered in the IBM SPSS Statistics 23 Statistical Package according to the questionnaire’s guide [29]. The import of SF-36 questionnaire data was done using the special software Quality Metric Health Outcomes Scoring Software 5.0, which was provided free of charge by OPTUM. ANOVA was used statistical analysis in order to see the difference in both the physical dimension and the mental dimension of the tool, for the social status indicators (education, income and occupation). For the variable smoking, 3 categories were initially created (smokers, ex-smokers and non-smokers). Then, for a better evaluation of the smoking habit, the variable Pack-years of smoking was created, which was calculated with the following equation: pack-year of smoking = Years that someone smokes or smoked × (number of cigarettes per day) / 20. For the rest (non-smokers) the value is 0.

Linear regression was also used to calculate the correlation coefficient of movement at the different levels of the variables in relation to the quality of life (2 dimensions). Regression models did not account for clustering at the neighbourhood level due to the small number of participants per neighbourhood, and thus, differences in health-related quality of life were only explored across quartiles of increasing/decreasing socio-economic disadvantage.

## Results

The survey response rate was 85.9%, which is very high. This is due to a previous postal communication (distribution of an open letter to all homes on the default streets) informing potential participants that a researcher from the university will follow up for a short interview in the next few days, as well as the importance and contribution of the study, with a request to participate.

### Demographics

The participants were 50% (*N* = 225) and 50% women (*N* = 225). The majority were 93.3% (*N* = 420) of Cypriot nationality, while the mean age of the sample was 53.7 ± 6.54. The results showed that 84% (*N* = 378) of the sample were either married or cohabiting, while2.7% (*N* = 12) stated to be single.

### Social status

The majority of the participants 80.8% (*N* = 364) has secondary upper and tertiary education Furthermore, 73.3% (*N* = 330) of the participants are employed either under full-time (68.4%) or part-time (4.9%). The family’s monthly income seems to vary, 7.3% have a family monthly income of over € 5000, while, 26.4%, has between 1500 and 2000 euros.

### Health behaviours

Participants were asked regarding smoking, alcohol and physical activity. More than half of the participants 57.8% (*n* = 260) reported to be non-smokers, while 35.8% (*n* = 161) consisting of smokers. it appears that the participants consume alcohol in their socialization, since almost half of them (41.6%, *n* = 187) reported consuming alcohol monthly. In relation to physical activity, 48% intense physical activity. When participants were asked to evaluate their health (self-rated health), 39.3% (*n* = 177) reported excellent or very good, 44% (*n* = 198) good and 16.7% (*n* = 75) moderate or poor.

### Quality of life

**Fig. 1:**
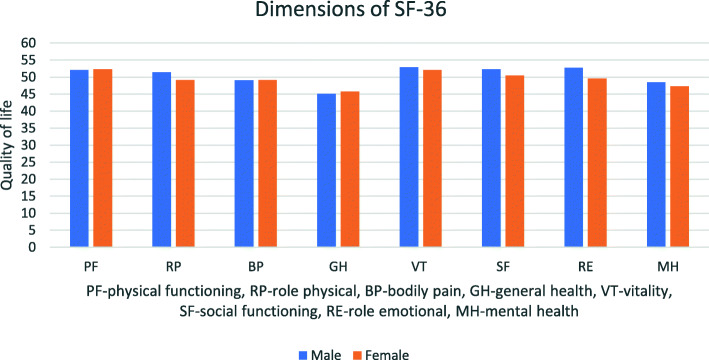
SF-36 dimensions in relation to gender

Females seem to have lower scores in both dimensions of the SF-36. In mental dimension, differences between the gender seems to be greater, because men have higher average values for all variables of mental dimension (Fig. 1). Scores ranged from 45 to 53.

### Model of social status using individual characteristics and gender interaction

The mean values for quality of life in physical and mental dimension were 49.7 (S.D. = 7.7) and 51.3 (S.D. = 7) for men. The corresponding values for female gender are 50 (S.D. = 8.5) and 48.8 (S.D. = 7.9). Female gender seems to get lower in both dimensions of the SF-36. Especially in mental dimension, differences between the sexes seems to be stronger, because men have higher average values for all variables of the mental dimension.

**Fig. 2:**
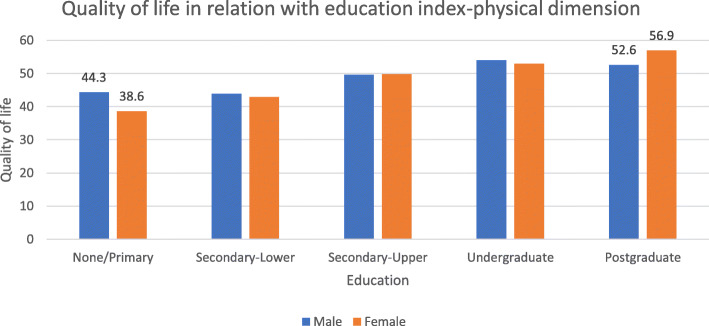
Quality of life in relation with education index-physical dimension

The social gradient seems to be appeared in all social indicators (Table 1). As for the physical dimension of health, it seems that a strong relationship exists between quality of life with the education index. Specifically, the difference between the two poles of the socio-economic scale (1st quartile and 4th quartile) is 12 for men and 14 for women. However, the result is not statistically significant. The value of the statistical control is 0.2 (Fig. 2). As for the mental health dimension, the pattern of social gradient is evident, but the image is not so strong. In this dimension the difference between the poles is almost 5 points for men and 9 for women (Table 3). Scores ranged from 38 to 54.

**Table 1:** Interaction of education, income and classification of occupation with quality of life - Physical dimension

PCS	Overall		Male		Female	
Education Level	Ν (Number)	Μ (SD) Mean (standard Deviation)	Ν (Number)	Μ (SD) Mean (standard Deviation)	Ν (Number)	Μ (SD) Mean (standard Deviation)
None/Primary	26	41,95 (10,86)	15	44,35 (9,49)	11	38,68 (12,18)
Secondary-Lower	56	43,46 (9,37)	26	43,99 (9,09)	30	42,99 (9,74)
Secondary-Upper	191	49,78 (7,02)	104	49,68 (6,83)	87	49,89 (7,28)
Undergraduate	146	53,05 (6,11)	63	53,09 (6,21)	83	53,03 (6,07)
Postagraduate	27	54,89 (5,35)	13	52,63 (6,40)	14	56,99 (3,10)
*P*-value		< 0,001		< 0,001		< 0,001
Per category increase (95% C.I.)		3,81 (3,08-4,53)		3,04 (2,03–4.05)		4,58 (3,55-5,62)
P for trend		< 0.001		< 0.001		< 0.001
P for interaction						0,16
Income (€)	**Ν**	**Μ (SD)**	**Ν**	**Μ (SD)**	**Ν**	**Μ (SD)**
< 1000	63	42,61 (9,94)	21	42,11 (8,89)	42	42,86 (10,52)
10,001–1500	117	48,55 (7,82)	59	47,39 (7,70)	58	49,73 (7,82)
1501–2000	119	51,16 (7,33)	64	50,78 (7,79)	55	51,61 (6,79)
2001–2500	77	53,19 (5,29)	40	53,13 (5,17)	37	53,25 (5,50)
3001–5000	40	52,25 (6,22)	20	51,83 (5,64)	20	52,68 (6,87)
> 5001	33	52,80 (6,61)	21	51,92 (5,80)	12	54,33 (7,86)
P-value		< 0,001		< 0,001		
Per category increase (95% C.I.)		2,00 (1,50-2,50)		1,84 (1,16-2,52)		2,23 (1,49-2,97)
P for trend		< 0.001		< 0.001		< 0.001
P for interaction						0,92
Classification of occupation	**Ν**	**Μ (SD)**	**Ν**	**Μ (SD)**	**Ν**	**Μ (SD)**
Level 1	17	48,31 (7,70)	7	49,86 (3,96)	10	47,22 (9,58)
Level 2	123	51,03 (6,91)	44	49,97 (7,90)	79	51,63 (6,27)
Level 3	99	51,97 (5,90)	81	51,44 (5,78)	18	54,34 (5,99)
Level 4	71	53,84 (5,06)	35	53,91 (4,27)	36	53,77 (5,78)
P-value		0,002		0,04		0,01
Per category increase (95% C.I.)		1,51 (0,72-2,31)		1,68 (0,50-2,86)		1,58 (0,46-2,71)
P for trend		< 0,001		0,005		0,006
P for interaction						0,31

The pattern of social gradient exists when income is concerned. The physical component of the model shows a strong relationship between health-related quality of life and income, both in men and women (*p* < 0.001). For women, the difference between the two poles is almost 11.5 points, while for men almost 10. The increase per category is 2.2 points for women and 1.8 for men. No differentiation is observed between genders (p for interaction = 0.9) (Table 1).

Regarding the classification of occupation, this seems to vary, since the relationship of the occupational status, irrespective of the way of classification, occupation seems systematically to present stronger relationship in men than in women, both in physical and mental dimensions. The difference between the poles in the physical dimension of the tool is 13 in men and 10 in women. (Tables 1 and 2).

**Table 2:** Interaction of education, income and classification of occupation with quality of life – Mental dimension

MCS	Overall		Male		Female	
Education Level	Ν (Number)	Μ (SD) Mean (standard Deviation)	Ν (Number)	Μ (SD) Mean (standard Deviation)	Ν (Number)	Μ (SD) Mean (standard Deviation)
None/Primary	26	46,18 (11,04)	15	47,95 (10,77)	11	43,77 (11,45)
Secondary-Lower	56	49,30 (8,93)	26	49,73 (7,37)	30	48,91 (10,20)
Secondary-Upper	191	49,59 (7,35)	104	51,22 (6,71)	87	47,63 (7,64)
Undergraduate	146	51,25 (6,50)	63	52,70 (6,00)	83	50,15 (6,68)
Postagraduate	27	52,61 (5,89)	13	52,45 (7,51)	14	52,75 (4,17)
P-value		0,005		0,10		0,015
Per category increase (95% C.I.)		1,40 (0,66-2,14)		1,32 (0,34-2,30)		1,65 (0,56-2,74)
P for trend		< 0,001		0,008		0,003
P for interaction						0,56
Income (€)	**Ν**	**Μ (SD)**	**Ν**	**Μ (SD)**	**Ν**	**Μ (SD)**
< 1000	63	43,88 (10,80)	21	43,79 (10,36)	42	43,92 (11,14)
10,001–1500	117	49,53 (7,13)	59	50,35 (7,36)	58	48,70 (6,85)
1501–2000	119	51,24 (6,16)	64	52,09 (5,84)	55	50,26 (6,44)
2001–2500	77	51,90 (6,32)	40	53,58 (4,95)	37	50,09 (7,15)
3001–5000	40	53,19 (4,40)	20	54,49 (3,71)	20	51,89 (4,74)
> 5001	33	51,91 (5,44)	21	51,83 (6,02)	12	52,04 (4,49)
P-value		< 0,001		< 0,001		< 0,001
Per category increase (95% C.I.)		1,56 (1,10-2,04)		1,40 (0,77-2,04)		1,57 (0,86-2,29)
P for trend		< 0,001		< 0,001		< 0,001
P for interaction						0,72
Classification of occupation	**Ν**	**Μ (SD)**	**Ν**	**Μ (SD)**	**Ν**	**Μ (SD)**
Level 1	17	51,31 (5,52)	7	52,98 (2,44)	10	50,15 (6,83)
Level 2	123	48,80 (8,00)	44	50,89 (7,67)	79	47,63 (7,99)
Level 3	99	52,70 (4,53)	81	53,02 (4,18)	18	51,23 (5,76)
Level 4	71	51,51 (5,70)	35	52,73 (5,48)	36	50,32 (5,73)
P-value		< 0,001		0,22		0,11
Per category increase (95% C.I.)		1,17 (0,34-2,01)		0,67 (−0,40, 1.75)		1,01 (−0.25, 2.27)
P for trend		0,006		0,22		0,11
P for interaction						0,90

### Model of social status using characteristics of the community/household (indicators of socio-economic disadvantage) and gender interaction

The socio-economic disadvantage indicators were grouped into two categories. The first category *Educational and socio-economic disadvantage* includes the variables: Education, Non-Cypriot nationals, Non-Cypriot non-European citizens, Single parent households, Middle age, Households over 5, Unemployment, Not owner occupied and elementary agriculture occupations. The second category *Structural disadvantage* includes the variables: Housing units pre 1980, Housing units post 2001, Vacant temp houses, Apartment blocks and Apartment mixed use.

#### Physical dimension

The variable of education seems to be clearly presented the social gradient. The increase per category, in the whole sample, is 1.23 points and is statistically significant (*p* < 0.001). The relationship between men and women differs. Specifically, in males the difference between the extremities (1st quadrant and 4th quadrant) is about 2 points, whereas in women this difference is around 6 points, and although it is not statistically significant (*P* = 0.15), it is clinically significant on the scale of SF36. Τhe other variables also present the social gradient, with no gender differences.

The variable *apartment blocks* presents the social gradient, but in reverse. According to this variable, people who live in neighbourhoods with more apartment blocks (4th quarter) have better quality of life. The increase per category across the sample is 0.87 and the pattern does not differ between men and women.

#### Mental dimension

Regarding the mental dimension, it does not seem to be any gender differentiation (Table 2).

### Social class and health behaviors

#### Income

Smoking seems not to be related to the social status of a person (Table 3). Those with the highest smoking status> 50 pack years of smoking (15.2%) do not appear to differ significantly from those to the lowest social position (20.6%).

**Table 3:** Social class (income, education, classification of occupation) and health behaviors

	Income						Education					Classification of occupation			
	< 1000	1001–1500	1501–2000	2001–2500	3000–5000	> 5000	None Primary	Secondary-Lower	Secondary-Upper	Undergraduate	Postgraduate	Level 1	Level 2	Level 3	Level 4
**Smoking**							42.3%	51.8%	55%	68.5%	74.1%				
< 1 pack years of smoking	55.6%	52.1%	64.7%	62.3%	67.5%	54.5%	0%	14.3%	10.5%	13%	7.4%	35.3%	60.2%	50.5%	70.4%
1–25 pack years of smoking	9.5%	12%	7.6%	10.4%	17.5%	15.2%	26.9%	16.1%	19.9%	11.6%	11.1%	17.6%	13%	8.1%	14.1%
> 25 > 50 pack years of smoking	14.3%	20.5%	16%	14.3%	15%	15.2%	30.8%	17.9%	14.7%	6.8%	7.4%	35.3%	17.9%	25.3%	7%
> 50 pack years of smoking	20.6%	15.4%	11.8%	13%	0%	15.2%	0.006					11.8%	8.9%	16.2%	8.5%
p- value	0.36											0.02			
**Alcohol consumption**							7.7%	3.6%	2.6%	4.1%	0%				
Often or daily	1.6%	3.4%	4.2%	1.3%	5%	6.1%	3.8%	14.3%	10.5%	8.2%	7.4%	0%	4.1%	6.1%	1.4%
1–2 times a week	7.9%	10.3%	12.6%	9.1%	5%	6.1%	23.1%	12.5%	20.9%	30.1%	18.5%	11.8%	12.2%	8.1%	12.7%
2–3 times a month	15.9%	21.4%	26.1%	31.2%	20%	21.2%	30.8%	19.6%	46.1%	44.5%	55.6%	35.3%	24.4%	25.3%	25.4%
Almost once a month	31.7%	33.3%	46.2%	44.2%	50%	54.5%	34.6%	50%	19.9%	13%	18.5%	29.4%	45.5%	45.5%	47.9%
Hardly any or no	42.9%	31.6%	10.9%	14.3%	20%	12.1%	< 0.001					23.5%	13.8%	15.2%	12.7%
p- value	0.002											0.85			
**Physical activity**							69.2%	61.8%	47.4%	39%	40.7%				
Low intensity	69.4%	48.3%	42%	44.2%	35%	51.5%	23.1%	23.6%	31.6%	48.6%	48.1%	47.1%	41.5%	47.5%	38%
Moderate intensity	22.6%	34.5%	39.5%	39%	47.5%	36.4%	7.7%	14.5%	21.1%	12.3%	11.1%	29.4%	37.4%	31.3%	47.9%
Vigorous intensity	8.1%	17.2%	18.5%	16.9%	17.5%	12.1%	0.001					23.5%	21.1%	21.2%	14.1%
p- value	0.07											0.43			

Regarding the frequency of alcohol consumption, it seems that people with higher income (higher social status) consume alcohol often or even daily (6.1%), compared to people with lower social status who consume alcohol at the same frequency (1.6%) (*p* = 0.002).

Physical activity did not appear to be related to income (*p* = 0.07).

#### Education

Education was statistically significant across all health behaviors investigated in this study. Compared to those with postgraduate education level who smoke> 50 pack years of smoking (7.4%), those with none/primary education (30.8%) are significantly much more (*p* = 0.006) (Table 3).

The same picture is seen in the frequency of alcohol consumption, where people with none/primary education (7.7%) are much more, than people with postgraduate education (0%) (*p* < 0.001).

Physical activity was statistically significantly correlated with education level (*p* = 0.001). People with secondary-upper education level appear to have more physical activity (21.1%), than those with none/primary education (7.7%).

#### Classification of occupation

The occupation classification is statistically significant with smoking (*p* = 0.02) (Table 4).

**Table 4:** *Occupation classification and health behaviors*

	Occupation classification			
	Level 1	Level 2	Level 3	Level 4
**Smoking**				
< 1 pack years of smoking	35.3%	60.2%	50.5%	70.4%
1–25 pack years of smoking	17.6%	13%	8.1%	14.1%
> 25 > 50 pack years of smoking	35.3%	17.9%	25.3%	7%
> 50 pack years of smoking	11.8%	8.9%	16.2%	8.5%
p- value	0.02			
**Alcohol consumption**				
Often or daily	0%	4.1%	6.1%	1.4%
1–2 times a week	11.8%	12.2%	8.1%	12.7%
2–3 times a month	35.3%	24.4%	25.3%	25.4%
Almost once a month	29.4%	45.5%	45.5%	47.9%
Hardly any or no	23.5%	13.8%	15.2%	12.7%
p- value	0.85			
**Physical activity**				
Low intensity	47.1%	41.5%	47.5%	38%
Moderate intensity	29.4%	37.4%	31.3%	47.9%
Vigorous intensity	23.5%	21.1%	21.2%	14.1%
p- value	0.43			

## Discussion

### Main results

Compared to other studies [12, 30–33], that determine the social status of the individual with individual characteristics, the currrent study investigated the quality of life, both in terms of individual characteristics and characteristics of the community/household (indicators of socio-economic disadvantage).

### Social status using individual’s characteristics and health-related quality of life

As in other studies, this study appears to have a strong correlation between demographic characteristics and health-related quality of life [15, 34–38]. In particular, a strong correlation appears to be associated with variables age (younger), level of education (older), income (older) and employment status (full-time). The phenomenon of the healthy worker would explain to some extent the correlation observed in the quality of life. Τhe healthy worker effect refers to the consistent tendency of active workers have a more favorable mortality experience than what the general population [39]. According to this phenomenon, workers usually have lower overall death rates than the general population, because seriously ill and chronic disabilities are usually excluded [40], from employment.

The results of the study show that women have lower values in all health-related quality of life indicators. These results are consistent with other studies in the literature showing that men receive higher average health-related quality of life than women [15, 34, 41, 42]. Women are likely to experience multiple roles. The expectations others have of women in any of the multiple roles, may differ from the expectations of themselves and come into conflict with their goals as individuals [43].

### Social status using characteristics of the community/household (indicators of socio-economic disadvantage) and quality of life

Indicators with the most statistical significance that reflect social gradient is the education level (*p* = < 0.001), unemployment (*p* = 0.001) and the absence of a computer (*p* = 0.001). Specifically, people who live in neighbourhoods where the education level is higher, unemployment is lower and most homes have a computer, have higher levels of quality of life. The above findings are consistent with studies investigating the health-related quality of life in relation to the socio-economic level [36].

### Interaction of social status and gender

The exploration of social status (individual indicators) in relation to health-related quality of life was conducted using the variables of education level, income and classification of occupation. In contrast to other studies [12, 30–32], which do not show the social inclination and how this differs between men and women, the phenomenon of social gradient is clear to the whole population, both for both- physical and mental dimension of the tool.

In an attempt to explore whether the pattern is changing by studying both genders separately, it has been found that the pattern of social gradient remains, with women having a stronger relationship with the variable level of education and income, whereas this relationship appears to be reversed in the variable occupation, where men present the strongest relationship.

### Social status and health behaviors

To some extent, health-related quality of life seems to be related to health behaviors, but health behaviors cannot fully explain this association. Health behaviors are not related to the observed gradation in the health-related quality of life of individuals.

### Comparison with other countries

**Fig. 3:**
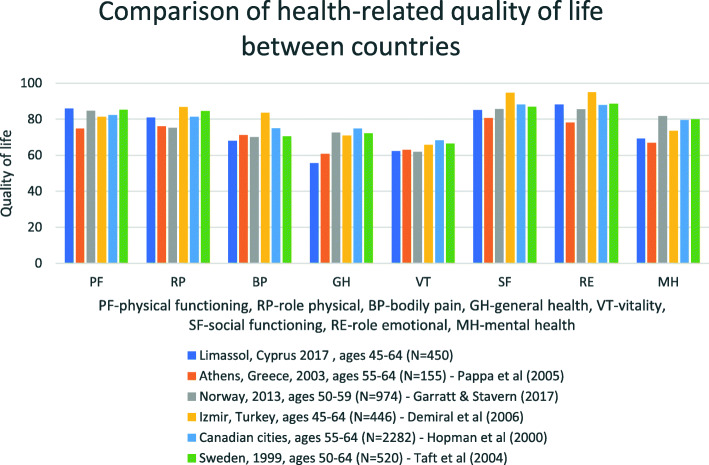
Comparison of health-related quality of life between countries

Compared to other selected studies in the literature, overall health-related quality of life indicators appear to be lower in Limassol. This is the case for both the physical dimension indicators and the mental dimension indicators. Athens (Greece) is the only exception. Looking at the populations of Limassol and Athens individually, appear to have a very similar picture in the 8 indicators of the tool (Fig. 3). Scores ranged from 55 to 95.

### Strengths of the analysis

This study is ground-breaking for Cypriot standards since is the first time, that data from 45 neighbourhoods (streets) of Limassol are presented, regarding the size and the extent of social inequalities in health-related quality of life and health behaviors.

The results of the study can be used to inform and guide prevention strategies and programs aimed at reducing health inequalities.

This study explores the impact of social inequalities in health in a single city, but it is the starting point for raising awareness among other researchers about future studies with new research questions on health-related quality of life. In addition, it is the trigger for the study of other cities, beyond the city of Limassol.

In addition, this survey will raise the awareness of health professionals, especially those working in the community, in regards to the impact of social inequalities on health-related quality of life.

### Limitations

This study has been conducted only in the city of Limassol. However, it is the first study to give population norms for Cyprus. Unlike other countries, in Cyprus there is no ranking of the professions. For this purpose, the authors adopted the Standard Occupational Classification of United Kingdom.

In the current study, the standard values of the general population of the United States of America were used, considering gender, as there are no standard rates for the general population of Cyprus.

Regarding the decision to interview only during the afternoon hours, there was a bias. Those who did not meet the criteria were excluded.

The results were not adjusted for age because this survey has a specific age group as target (45–65 years old).

## Conclusions

Exploring social inequalities in quality of life, is a complex state influencing social physical and psychological state of health. According to Wanderley [44], there is not a single study or tool capable of simultaneously clarifying the mechanisms identified as related to health and functionality (e.g. living conditions, financial status, marital status, lifestyle).

It seems that being male, young, highly educated, with high income, working full time and having a mild physical activity, results in a significant higher level of quality life in relation to others.

With respect to the characteristics of the community/household associated with social status, the results show that low levels of education, unemployment and the absence of a computer at home are significantly associated with low levels of quality of life. However, there is no indication to support any differentiation between men and women. Regarding linear regression with respect to individual’ s characteristics, the study showed that strong predictors associated with low health-related quality of life are all three individual characteristics of social status- education level, income and occupation. This relationship seems to be stronger in women.

Some studies show that gender affects the patterns of risk factors and that this has a different impact on quality of life. Therefore, gender specificities must be taken into account in health prevention strategies [12, 33].

## Supplementary Information

**Additional file 1.**

## Data Availability

The datasets used and analyzed during the current study are available from the corresponding author on reasonable request.

## Abbreviations

HRQol: Health-related quality of life
