# Supplementary material for: Social gradient in health-related quality of life among urban middle-age residents in Limassol, Cyprus: research article
Source: BMC Public Health. 2021 Mar 29;21:608. doi: 10.1186/s12889-020-10027-6 (PMC8008686; doi:10.1186/s12889-020-10027-6)
Supplement: Supplementary file 1 — Additional file 1. [file 12889_2020_10027_MOESM1_ESM.docx]

**Appendix 1**

**Demographic / socio-economic characteristics questionnaire**

| **1.** | Gender | Male Female | | | **2.** Age | | .................... years | | | | | | |  | | |
| --- | --- | --- | --- | --- | --- | --- | --- | --- | --- | --- | --- | --- | --- | --- | --- | --- |
| **3.** | Nationality | Cypriot | | | Other …….……………….… | | | | | | | | | | | |
| **4.** | Marital status | Married | | | Single | | Divorced/  Separated | | | Widow | | | In cohabitation | | | |
| **5.** | Total number of family children | ……………  Children | | | **6.** Number of family members living in the same house | | | | | ……………  people | | | | | | |
| **7.** | Education | None | Primary | | Secondary-Lower | Secondary-Upper | | | Undergraduate | | | | Postgraduate | | |  |
| **8.** | Employment status | Full time | | | Part time | Unemployed | | I do not work | | | | | **9.** Occupation  ……………….. | | | |
|  |  |  | | |  |  | |  | | |  | |  | |  | |
| **10.** | Family net monthly income | <1000 | | | 1001-  1500 | 1501-  2000 | | 2001-2500 | | | 2501-3001 | | 3001-5000 | | 5001 και άνω | |
| **11.** | In the last 12 months, have you had any difficulties with the household's current expenses, such as paying bills, buying basic necessities, etc.? | | | | | | | | | | | | Yes | | ‘No | |
| **12.** | How long have you been living at this address? | Less than 1 year | | | 1-3 years | | 3-5 years | | | 5-10 years | | | Over 10 years | | | |
| **13.** | Do you live in a privately owned or rented house? | Owed | | | Rented | | Other …………………………….. | | | | | | | | | |
| **14.** | House type | Detached house | | Semi-detached house | | Apartment building with less than 8 apartments | | | | | | Apartment building with over than 8 apartments | | | | |

1. **How would you self-rate your general state of health?**

|  | Excellent |
| --- | --- |
|  | Very good |
|  | Good |
|  | Moderate |
|  | Poor |

1. **Do you smoke;** Yes No

**16.1. If you answered YES above, please answer the questions below**

How many years do you smoke? ...........................

About how many cigarettes do you smoke a day?..........................

**16.2. If you answered NO, please answer the questions below**

Have you been smoking in the past? Yes NO

YES, for how many years in total did you smoke? ……………………

How many cigarettes did you smoke a day? ………………..

1. **Alcohol: in the last 12 months, how often have you consumed alcohol (beer, wine, alcoholic beverages, a cocktail containing alcohol)?**

|  | Everyday |  | 2-3 per month |
| --- | --- | --- | --- |
|  | 5-6 times per week |  | Once per month |
|  | 3-4 times per week |  | 3- 11 times per year |
|  | 2 times per week |  | Less than 2 times per year |
|  | Once per week |  | Never |

Think of a typical day to drink: approximately how many alcoholic beverages do you consume?

1 drink 2 drinks 3-4 drinks 5-6 drink 7-9 drinks over 10 drinks
